# Endocrine organs of cardiovascular diseases: Gut microbiota

**DOI:** 10.1111/jcmm.14164

**Published:** 2019-01-27

**Authors:** Qiujin Jia, Yingyu Xie, Chunmiao Lu, Ao Zhang, Yanmin Lu, Shichao Lv, Junping Zhang

**Affiliations:** ^1^ First Teaching Hospital of Tianjin University of Traditional Chinese Medicine Tianjin China; ^2^ Tianjin University of Traditional Chinese Medicine Tianjin China; ^3^ Epidemiology College of Global Public Health New York University New York New York; ^4^ Tianjin Nankai Hospital Tianjin China

**Keywords:** cardiovascular diseases, gut microbiota, metabolites, risk factors, treatment

## Abstract

Gut microbiota (GM) is a collection of bacteria, *fungi, archaea, viruses and protozoa*, etc. They inhabit human intestines and play an essential role in human health and disease. Close information exchange between the intestinal microbes and the host performs a vital role in digestion, immune defence, nervous system regulation, especially metabolism, maintaining a delicate balance between itself and the human host. Studies have shown that the composition of GM and its metabolites are firmly related to the occurrence of various diseases. More and more researchers have demonstrated that the intestinal microbiota is a virtual ‘organ’ with endocrine function and the bioactive metabolites produced by it can affect the physiological role of the host. With deepening researches in recent years, clinical data indicated that the GM has a significant effect on the occurrence and development of cardiovascular diseases (CVD). This article systematically elaborated the relationship between metabolites of GM and its effects, the relationship between intestinal dysbacteriosis and cardiovascular risk factors, coronary heart disease, myocardial infarction, heart failure and hypertension and the possible pathogenic mechanisms. Regulating the GM is supposed to be a potential new therapeutic target for CVD.

## 1 THE ROLE OF gut microbiota IN HOST PHYSIOLOGY

Many microbes inhabit the human gastrointestinal tract including viruses and bacteria, fungi and protists, which make up the intestines’ symbiotic microbes.^1^ The number of bacteria carried by the human body is approximate to about 10^14^, mostly including anaerobes and the composition of the gut microbiome is similar up to the phylum level (mainly *Bacteroidetes* and *Firmicutes*), but the diversity and richness of species are variable between individuals.^2^, ^3^, ^4^, ^5^ These bacteria can be roughly classified into three categories according to their effects on the human body: (a) Physiological bacteria (symbiotic with the host), such as *Bifidobacterium, Lactobacillus*. (b) Conditional pathogens, such as *Enterobacteriaceae, Enterococcus*. (c) Pathogens, such as *Proteus, Staphylococcus aureus*. The microbiota in the gut has many crucial functions in human health and affects the host via various distinct pathways. Studies have shown that the gut microbiota (GM) is directly involved in the body's nutrient absorption, growth and development, biological barriers, immune regulation, metabolism and many other aspects.^6^, ^7^, ^8^

## 2 METABOLITES AND EFFECTS OF GM

Metabolites produced by GM include trimethylamine‐N‐oxide (TMAO), bile acids (BAs), short‐chain fatty acids (SCFAs), protocatechuic acid (PCA) and other metabolites, such as phenylacetylglutamine, p‐cresyl sulfate, indoxyl sulfate (IS), enterolactone and H_2_S.

### 2.1 Trimethylamine‐N‐oxide

Trimethylamine‐N‐oxide is a biologically active molecule and a putative promoter of chronic diseases including atherosclerosis in humans.^9^ In the human body, TMAO is established as trimethylamine (TMA). TMA comes directly from foods rich in choline, lecithin and L‐carnitine, such as red meat, eggs, dairy products and saltwater fish.^10^, ^11^ Most of TMA is absorbed into the bloodstream and then rapidly oxidized to TMAO by flavin‐containing monooxygenase‐3 (FMO3), a hepatic enzyme.^10^ TMAO can affect lipid metabolism causing accumulation of cholesterol in cells and it can also have a direct impact on platelet function, promote an inflammatory response and so on. The effect of the TMAO will accelerate the development of atherosclerosis and the level of plasma TMAO and its associated metabolites are directly proportional to the risk of cardiovascular disease (CVD), which will be pointed out in the following sections.^12^, ^13^, ^14^, ^15^, ^16^

### 2.2 Bile acids

Bile acids are amphipathic molecules synthesised from cholesterol in the liver. This process is an important way to eliminate cholesterol from the human body. After eating, BAs stored in the gallbladder are secreted into the intestine to assist the emulsification of dietary fats and assist the intestinal absorption of lipid nutrients and lipid‐soluble vitamins.^17^, ^18^ BA itself regulates metabolism, mainly through two receptors, namely the nuclear farnesyl X receptor (FXR) and the G protein‐coupled receptor TGR5.^17^, ^19^ BAs can control intestinal bacterial overgrowth, in turn, intestinal bacteria can regulate host metabolism by metabolism BAs.^20^ Recent studies have shown that BA has a pleiotropic and hormonal activity that regulates lipid and glucose metabolism, controls inflammation and fibrosis, maintains vascular integrity, restores the intestinal barrier and controls ageing and circadian rhythms.^19^ Further studies have confirmed that FXR activation can reduce intestinal ischemia‐reperfusion injury and preserve internal structure and permeability. Also, FXR agonists decrease the release of pro‐inflammatory cytokines, reduce autophagy inhibition and modulate obesity and related metabolic phenotypes.^21^, ^22^

### 2.3 Short‐chain fatty acids

Short‐chain fatty acids primarily originate from the fermentation of dietary fiber in the gut and they are subsequently absorbed into the bloodstream of the host, in which they interact with host proteins, thereby affecting host physiology.^23^ Acetate, propionate and butyrate (roughly 60:25:15 in the colon) account for 80% of the SCFA produced by intestinal microflora.^24^ In addition to their important role as fuel for colonic epithelial cells, SCFAs also modulate different cell signal transduction processes via G protein‐coupled receptors GPR43 and GPR41.^23^, ^25^ Recent evidence has suggested that SCFAs also play an important role in glucose and lipid metabolism and butyrate has been demonstrated to decrease LPS translocation, inhibit macrophage activation, thus reducing the production of inflammatory factors and reactive oxygen species (ROS) production.^26^, ^27^, ^28^, ^29^ SCFAs can also inhibit the inflammatory reaction by reducing the migration and proliferation of immune cells, reducing various cytokines and inducing apoptosis. Therefore, SCFA is considered to have an anti‐inflammatory effect.^30^

### 2.4 Protocatechuic acid

Protocatechuic acid is chemically known as 3,4‐dihydroxybenzoic acid and is one of the main metabolites of complex polyphenols such as anthocyanins and procyanidins that are normally found in high concentrations in vegetables and fruit, such as onions, plums, gooseberries and grapes.^31^, ^32^ More and more evidence support that PCA can play diverse biological effects by acting on different molecular targets, such as antioxidant, anti‐inflammatory and anti‐hyperglycemic and neuroprotective activity.^32^ PCA has been implicated in the progression of atherosclerosis. Some scholars concluded that PCA could inhibit OA‐induced vascular smooth muscle cells proliferation, improve the endogenous antioxidant capacity of macrophages and promote cell proliferation and cell survival via IGF‐I signalling.^33^, ^34^, ^35^ Besides, studies have demonstrated that PCA can improve cardiac function and cardiac autonomic balance, prevent cardiac mitochondrial dysfunction and increase anti‐apoptotic protein.^36^ In a recent experimental study on Type 2 diabetes mouse models it was found that, PCA could significantly stimulate glucose metabolism in skeletal muscle, regulate glycemic and lipid status, reduce the secretion of pro‐inflammatory cytokines.^37^

### 2.5 Phenylacetylglutamine, P‐cresyl sulfate, IS, enterolactone and H_2_S

**Figure 1:**
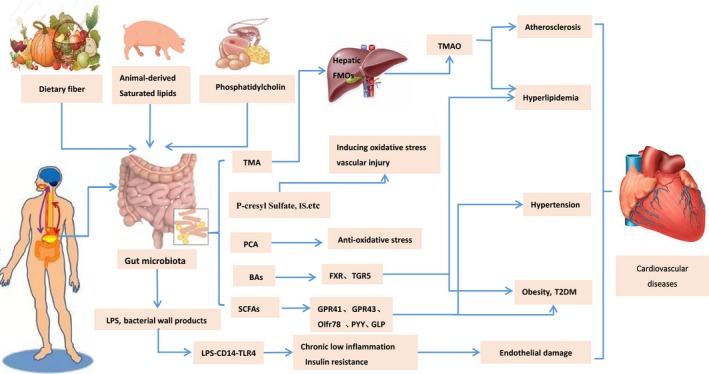
Gut microbiota and its metabolites linked to cardiovascular diseases

Proteins in the intestine are metabolized by harmful microorganisms (such as Clostridium perfringens) to produce harmful amines, phenols and ammonia, causing human diseases and discomfort, which have a great impact on the human cardiovascular system.^38^, ^39^ Studies have found that phenylacetylglutamine and p‐cresyl sulphateare lower in patients with non‐coronary heart disease and p‐cresyl sulphate is a significant independent predictor of carotid plaque burden.^40^ *Escherichia coli*‐based intestinal flora produce a large amount of quinone by metabolizing the tryptophan in food and further oxidize it to indophenol under the action of microbial oxidase. Similar to TMAO, indophenols in the liver are sulphonatedto form IS.^41^ Excessive accumulation of IS may affect oxidative stress in cardiomyocytes, induce myocardial cell damage and cause vascular endothelial cell damage to inhibit self‐repair and increase vascular access thrombosis.^42^ Enterolactone is produced primarily by intestinal digestion of fibre‐rich foods. In a meta‐analysis, it was found that the higher risk of acute coronary accidents was lower in patients with higher serum enterolactone and therefore the lower enterolactone may be another risk factor for coronary heart disease (CHD).^43^ In addition, some sulphate‐reducing bacteria in the human intestinal tract produce a large amount of H_2_S using sulphate as a substrate. Studies indicate that H_2_S acts as a gas transmitter in the human body. H_2_S is an essential mediator of various physiological processes in the human body including cell protection, vasodilation, angiogenesis, blood pressure regulation and heart rate reduction. It plays a major role in CVD (Figure 1).^44^, ^45^

## 3 INTESTINAL DYSBACTERIOSIS AND CARDIOVASCULAR RISK FACTORS

Hyperlipidaemia, obesity, diabetes and atherosclerosis are important risk factors for CVD. Plenty of evidence indicates that the GM is closely related to these four risk factors.

### 3.1 Hyperlipidaemia

A recent survey found that the GM composition can explain 6.0% of the variation in triglycerides and 4.0% of that in HDL‐C and 4.5% of that in BMI, independent of age, sex and genetics at the human population level.^46^ Studies have also found that individuals with low microbial richness have increased fasting triglycerides and decreased HDL‐C. Reverse cholesterol transport (RCT) is a key pathway involving the return of excess cholesterol from peripheral tissues to the liver to excretion of bile and eventually to faeces.^47^ In a recent article, a researcher found that TMAO reduced the expression of cholesterol 7‐alpha‐hydroxylase 1 (CYP7a1), the major BA synthetic enzyme in the catabolism of cholesterol, whose reaction is the rate‐limiting step. This finding may in part explain the impact of TMAO on RCT and upregulation of CYP7a1 has been reported to lead to increasing RCT and reduced atherosclerosis in susceptible mice.^48^, ^49^ Recent research shows that BA‐mediated activation of FXR inhibits the activity of CYP7a1 leading to RCT disorders and elevated cholesterol, but reduced triglycerides.^17^, ^50^ However, there are also studies showing that FXR knockout mice exhibit high‐density lipoprotein metabolism and RCT leading to elevated cholesterol.^51^ Recent studies have found that TMAO can cause cholesterol accumulation in cells by increasing the expression of the scavenger receptors, CD36 and SR‐A1 and the formation of foam cells.^16^, ^30^ In conclusion, intestinal flora metabolites are closely related to lipid metabolism, but its mechanism of action needs further investigation.

### 3.2 Obesity and type 2 diabetes mellitus

In 2013, Liou et al transferred caecal contents from RYGB donors to sterile mice and showed that the recipient mice showed significant weight loss indicating a correlation between GM and obesity.^52^ Study shows that *Enterobacter cloacae B29* is part of the direct cause of obesity, which is the first internationally recognized ‘fat bacteria.’^53^ Recent study indicates the characteristics of the flora associated with obesity, namely the increase in the number of *Firmicutes* and the reduction of *Bacteroides*. Similarly, this situation can occur in type 2 diabetes as well.^54^, ^55^ GM may cause obesity by reducing fasting‐induced adipokine factor (Fiaf) expression. Mandard et al^56^ found that Fiaf gene over‐expressing mice showed a 50% reduction in body fat storage compared to wild‐type mice. Intestinal flora can also stimulate the production of various inflammatory factors through the LPS‐CD14‐TLR4 pathway leading to chronic systemic inflammation and further cause obesity and insulin resistance. Animal study showed^57^ that high‐fat‐fed mice had increased LPS Gram‐negative bacteria in the intestine, elevated plasma LPS levels, causing metabolic endotoxemia and obesity and continued subcutaneous injection of LPS in mice also induced the above reaction. CD14 gene knockdown can reduce the inflammatory state of mice caused by LPS; TLR4 gene knockout can also prevent obesity and insulin resistance caused by high‐fat diet, thereby reducing obesity.^58^, ^59^ Obesity is often a leading factor associated with type 2 diabetes mellitus (T2DM), so the above mentioned mechanisms of intestinal flora causing obesity can also increase the risk of T2DM. By combining with GPR41 and GPR43, SCFA can promote intestinal L cells to secrete GLP‐1 and peptide YY (PYY). GLP‐1 can increase glucose‐dependent insulin secretion and promote islet cell proliferation. PYY can improve the survival and function of islet cells. Therefore, intestinal flora disorder can affect the occurrence and development of diabetes by affecting SCFA.^60^ There is evidence in other studies that activation of FXR and TGR5 mediated by BA can increase insulin sensitivity and regulate GLP‐1 secretion. However, its pathways are complex, many studies use animal models and the formation of a complete BA signalling pathway system needs further study.^61^ BA can also indirectly activate signal molecules such as inducible nitric oxide synthase, interleukin 18 (IL‐18) and fibroblast growth factor 19 (FGF‐19).^62^ Related study^63^ shows that FGF‐19 can improve glucose tolerance, reduce weight gain and increase the metabolic rate. So BA plays a major role in the occurrence and development of diabetes mellitus.

### 3.3 Atherosclerosis

Hyperlipidaemia is an independent risk factor for atherosclerosis. In the above discussion, we have discussed the effects of GM on lipid metabolism, which we will not state it here. In addition, studies have shown that TMAO can also directly affect platelet function and increase thrombosis risk. When platelets are directly exposed to high levels of TMAO, the intracellular storage of Ca2+ is released, which enhances platelet activation via a variety of agonists (thrombin, ADP, or collagen).^15^ There are also studies showing that TMAO enhanced the platelet reactivity and thrombosis risk and promoted vascular inflammation by activating the NLRP3 inflammasome.^16^ Using pyrosequencing to analyse the microbial species of atherosclerotic plaques and intestinal microflora in the same atherosclerotic patients, the results show that several bacteria exist simultaneously, suggesting that bacteria found in the plaque may be derived from intestinal flora.^64^ In another study using shotgun sequencing method it was found that atherosclerosis patients were rich in *Collinsell,* and healthy people were rich in *Roseburia* and *Eubacterium*.^65^ The above results suggest that the GM is directly related to the occurrence of atherosclerosis. In the above section, we have already mentioned that BA‐mediated FXR and SCFA, PCA have anti‐inflammatory effects and can regulate glucose metabolism; so the intestinal flora can also inhibit the development of atherosclerosis. However, the core mechanism that causes atherosclerosis remains to be further clarified.

## 4 GUT MICROBIOTA AND CARDIOVASCULAR DISEASE

### 4.1 Coronary heart disease

Coronary heart disease is one of the most common CVD in clinical practice, which is extremely harmful to human health. Some scholars have found that plasma TMAO levels are elevated in patients with coronary heart disease and the structure of intestinal flora is also changed and manifested by the decrease of *Lactobacillus* and *Bacillus*.^66^ A research showed that the structural changes in the GM could be used as a diagnostic marker for coronary heart disease.^67^ Using a high‐choline diet to feed ApoE‐/‐ C57BL mice, results display that higher the serum TMAO level in mice, larger was the area of the sizeable atherosclerotic plaque .^68^ Tang et al^69^ were followed up for 3 years in more than 4000 patients with coronary angiography and results showed that the concentration of plasma TMAO was positively correlated with the risk of the cardiovascular endpoint. Among all 2235 patients with coronary heart disease, the all‐cause mortality rate of TMAO (>6.5 mol/L) was 3.9 times higher than that of patients at a low level (<2.5 mol/L). At the same time, independent of the traditional predictors (hypersensitivity C reactive protein, myeloperoxidase, estimated glomerular filtration rate, etc.), TMAO can predict the long‐term risk of death in patients with coronary heart disease. From the perspective of metabonomics, the intestinal microflora metabolite TMAO indirectly proves the clinical prognostic value of intestinal microecology for coronary heart disease. TMAO can also be used to assess the degree of plaque burden of coronary heart disease.^70^ In short, the current research on TMAO is thorough and the level of TMAO is expected to become the standard for diagnosis and risk assessment of patients with coronary heart disease.

### 4.2 Myocardial infarction

Different from some other chronic CVD, acute myocardial infarction (AMI) is a critical stress injury to the body. Under this trauma, does the structure of the intestinal microbiota and the metabolites undergo stress changes? In myocardial infarction (MI) patients, typical intestinal flora disorders will occur, probiotics will decrease significantly, pathogenic bacteria will multiply in large numbers and a series of changes such as impaired intestinal barrier function and increased intestinal permeability will occur in the body. Analysing the blood flora of 49 healthy controls, 50 stable coronary heart disease and 100 ST‐segment elevations myocardial infarction (STEMI) patients, the researchers found that after STEMI, the abundance and diversity of blood flora were increased and more than 12% of the blood bacteria were from the intestinal flora. This indicates that a significant increase in intestinal flora in plasma is associated with systemic inflammation and cardiovascular adverse events after STEMI.^71^ Animal studies showed that AMI rats were parallel to intestinal barrier injury and intestinal flora richness was significantly higher than the sham group. Therefore, the intervention of intestinal flora structure to improve the clinical prognosis of AMI may be a new method of AMI treatment.^72^ Study has shown that probiotics can reduce cardiac hypertrophy in MI rats.^73^ Further study also showed that antibiotics could improve the area of MI in AMI mice, which may be related to the regulation of intestinal flora. However, the above study does not show if improvement in the structure of intestinal flora can prevent coronary atherosclerosis and reduce the incidence of AMI.^74^ In the MI model, left ventricular dysfunction and intestinal perfusion inadequacy, reduce tight junction protein expression, intestinal mucosa damage, increased intestinal permeability, antibiotic treatment to eliminate the intestinal bacterial translocation. It can reduce systemic inflammatory reaction and myocardial injury in model mice.^71^ Recently, Tang et al^75^ found that supplementation of SCFA can improve the physiology and survival of ABX (antibiotic‐treated mice) mice after MI and this study also found that MI caused a decrease in *Lactobacillus*. This indicates that the composition of intestinal flora and SCFA have obvious repair effect on heart tissue after MI. Therefore, the stability of intestinal flora may be of great significance to the rehabilitation of patients with MI.

### 4.3 Heart failure

As shown in recent studies, changes in intestinal microecology can directly damage cardiac muscle cells and cause cardiac dysfunction. In the mouse model of dietary intervention, it was found that elevated serum TMAO levels in mice resulted in cardiac injury and fibrosis and increased TMAO levels were prone to heart failure (HF).^76^ There are also studies which found that the levels of TMAO, choline and betaine were involved in left ventricular diastolic dysfunction.^77^ Also, studies found that, higher the TMAO level in patients with HF, higher the mortality rate in 5 years. Compared with the risk factors for HF, heart and kidney index and systemic inflammatory markers, TMAO can better evaluate the prognostic value.^78^ After a 1‐year follow‐up of 972 patients with acute HF, investigators found that serum TMAO levels could predict adverse prognostic events, whereas TMAO+NT‐proBNP could predict a higher value.^79^ A clinically controlled cohort study showed that serum TMAO levels were elevated in patients with chronic HF, and TMAO levels were associated with cardiac function and survival.^80^ Besides, chronic HF patients had significantly more pathogenic bacteria and *Candida* than healthy controls, which is a result concluded by multiple researchers. The inflammatory, intestinal permeability and right atrial pressure of these chronic HF patients were significantly increased and these were signals of venous congestion. Also, these correlations were stronger in patients with moderate to severe HF (NYHA heart function III‐IV) than those with mild HF (NYHA cardiac function I‐II).^81^ Therefore, improving intestinal flora and lowering TMAO level are both expected to improve the prognosis of patients with HF.

### 4.4 Hypertension

Recent findings show that SCFA bind the G protein‐coupled receptors GPR41 and GPR43 and olfactory receptors olfO78 in the kidney, heart, sympathetic ganglia and blood vessels to modulate blood pressure.^82^, ^83^ SCFA can induce the release of renin from the afferent arteriole and increase the blood pressure, which is mediated by Olfo78. This, in turn can be counteracted by the vasodilator action of GPR43.^82^, ^84^ GPR41 can increases energy expenditure by stimulating the sympathetic nervous system, but this could also lead to an increase in blood pressure.^85^ Other researchers have found that TMAO can increase blood pressure and hydrogen sulphate can directly act on blood vessels to modulate blood pressure.^86^, ^87^ Studies have shown that high salt may induce hypertension by inducing T helper 17 (TH17) cells, and *Lactobacillus* can reduce the number of TH17 cells and prevent the deterioration of salt‐sensitive hypertension.^88^ The *Firmicutes* and a *Bacteroidetes* ratio (F/B) was recently reported being increased in spontaneously hypertensive rats and AngII‐induced hypertension rats.^89^ Some scholars believe that GM facilitates AngII‐induced vascular dysfunction and hypertension, at least in part, by supporting an MCP‐1/IL‐17 driven vascular immune cell infiltration and inflammation.^90^ In addition, obstructive sleep apnoea (OSA) can increase the risk of systemic hypertension in individuals. Studies have demonstrated that intestinal flora imbalance may be a direct cause of OSA‐induced hypertension.^91^ There are also studies that confirm probiotics and their fermentation products have been shown in many studies to inhibit the production of nitrogen oxides in macrophages, reduce the species of ROS and increase the absorption of dietary calcium to lower blood pressure (Table 1).^92^

**Table 1:** Association of gut microbiota and related metabolites with cardiovascular disease

	Hyperlipidaemia	Obesity, T2DM	Atherosclerosis	Coronary heart disease	Myocardial infarction	Heart failure	Hypertension
Structural changes in the gut microbiota	*Enterobacteria*↑ *Probiotics*↓	*Firmicutes↑Bacteroides↓*	*Collinsella↑Roseburiam↓*	*Lactobacillus↓Bacillus↓*	*Probiotics*↓Pathogenic bacteria↑	*CandidaEscherichia coli*	*Firmicutes/Bacteroidetes* ratio↑
Major metabolites	TMAOFXR	SCFABAs	TMAOFXRPCA	TMAO H_2_SPhenylacetylglutamine,P‐cresyl sulphateIndoxyl sulphate, Enterolactone	SCFA	TMAOIndoxyl sulphate	SCFATMAOH_2_S
Possible mechanism of action	TMAO and FXR significantly inhibits RCT;CYP7a1↓CD36↑SR‐A1↑Foam cells↑Cholesterol↑	Chronic low‐grade inflammation and insulin resistance;LPS ↑Fiaf↓GLP‐1↓PYY↓FGF‐19↓	TMAO can directly affect platelet function and increase thrombosis risk;Cholesterol↑NLRP3 ↑FXR↓PCA↓	TMAO↑Abnormal glucose and lipid metabolism;InflammationPhenylacetylglutamine↑P‐cresyl sulphate↑Indoxyl sulphate↑ Enterolactone ↑	Gut permeability;Intestinal flora disorder;Lactate ↑LPS↑SCFA↓	TMAO↑Left ventricle diastolic dysfunction↑Myocardial fibrosis ↑	SCFA can regulate blood pressure with GPR41, GPR43 and Olfr78;Infusionof Ang II/TMAO associated with blood pressure;H2S can dilate blood vessels and reduce heart rate

## 5 THE ROLE OF GUT MICROBIOTA IN CARDIOVASCULAR DISEASE

Gut microbiota is an essential micro‐ecosystem in the human body and even some scholars believe it is the ‘virtual endocrine organ’ of the human body. Summarizing the above and combining current research progress, the mechanism of intestinal flora affecting CVD mainly includes three aspects including: (a) Intestinal flora disorder leads to bacterial endotoxin translocation and promotes the release of inflammatory factors leading to an inflammatory response; (b) intestinal flora disorder leads to abnormal metabolism of substances, which causes CVD such as lipid, glucose and tryptophan metabolism; (c) intestinal flora disorder promotes oxidative stress in the body and aggravates the development of CVD. As the first two mechanisms have already been discussed in the previous discussion. In next section, the discussion would be concentrating on oxidative stress.

Gut microbiota is also involved in the metabolism of purine and uric acid (UA). For example, xanthine dehydrogenase, the key enzyme responsible for the oxidative metabolism of purines is generated by the secretion of Escherichia coli in intestinal bacteria.^93^ Therefore, the decomposing activity of GM on UA is positively related to the content of *Escherichia coli*. The study found that the number of *E. coli* in patients with coronary heart disease increased, blood UA levels increased significantly, whereas high concentrations of UA showed prooxidation. Increased blood UA levels can lead to high level nitrite/nitrate in the blood, decreased bioavailability of NO and oxidative stress.^68^, ^94^ There are also studies showing high UA decreased cardiomyocyte viability and increased ROS production in cardiomyocytes.^95^ In addition, carotenoids, as antioxidants have an anti‐angina effect. The carotenoid genes were increased in healthy patients compared with arteriosclerosis patients.^65^ The GM disorder causes the decrease of the bacteria containing the synthetic carotenoid gene, the reduction of the level of carotenoid and the weakening of the antioxidant effect, thus promoting the development of AS. There have been studies that rigorously characterize the effect of probiotics on antioxidation regarding the intestinal microbiota composition. Probiotics may modulate the redox status of the host via their metal ion chelating ability, antioxidant systems, regulating signalling pathways, enzyme producing ROS and intestinal microbiota.^96^ In addition to the above mechanisms, intestinal microflora can also increase the level of nitric oxide through nitrate reduction in the non‐enzymatic pathway.^39^ At the same time, changes in the structure of GM, also affect the expression and activity of some other molecules acting on the blood such as the AMP‐activated protein kinase, hypoxia‐inducible factor‐1α and peroxisome proliferator‐activated receptor‐γ coactivator‐1α.^97^ These molecules can promote angiogenesis in skeletal muscle and other parts. Thus, intestinal microflora can not only affect the course of AS but also have potential effects on vascular dilatation, angiogenesis and blood supply.

## 6 THE TARGET OF FUTURE TREATMENT

At present, the improvement or reversal of intestinal microflora has become a hot spot for CVD, which includes the faecal microbiota transplantation, dietary regulation, probiotics, prebiotics, antibiotic intervention and so on.

### 6.1 Dietary regulation

Dietary regulation has been proven to be an effective strategy to reduce cardiovascular risk. The Mediterranean diet refers to the eating styles of vegetables, fruits, fish, Cereals, beans and olive oil in the southern European countries on the Mediterranean coast, such as Greece, Spain, France and southern Italy. This diet is especially popular in recent years and is confirmed to prevent CVD and reduce the mortality of CVD in men and women.^98^ Studies have shown that the level of TMAO in the urine of patients who did not adhere to the Mediterranean diet increased.^99^ In addition, high fibre diet and acetic acid (fibrous fermentation) can reduce the proportion of F/B, increase the number of bacteriobacterium, reduce myocardial fibrosis in hypertensive mice and prevent the progress of hypertension and HF.^100^

### 6.2 Probiotics and prebiotics

Probiotics in animals mainly include lactic acid bacteria, bifidobacteria, actinomycetes, yeasts and so on. Probiotics can inhibit inflammation, protect and repair the intestinal mucosal barrier and improve intestinal function. Probiotics are active microorganisms, which are beneficial to host health. The atherosclerotic plaque of the aorta was less than the control group after feeding the probiotic rhamnosus GG (LGG) to the ApoE‐/‐ mice fed with a high‐fat diet and various biomarkers including endotoxin were improved to some extent.^101^ Studies showed that after 3 months of treatment with Brucella, patients with chronic HF showed a decrease in creatinine, UA, hsCRP and a decrease in the diameter of the left atrium and a higher left ventricular ejection fraction (LVEF).^102^ Research has also shown that probiotics can directly absorb cholesterol and reduce the content of cholesterol in the medium and in the process of the growth of probiotics such as lactic acid bacteria, it can promote the metabolism of cholesterol, thus reducing the cholesterol content.^103^ Prebiotics are a dietary supplement (including isomalt oligosaccharides, oligosaccharides, Bifidus factors, etc.). They has a beneficial effect on the host by selectively stimulating the growth and activity of bacteria. A recent study has shown that probiotics can improve metabolic syndrome and infiltration of small intestinal cells.^104^ However, probiotics remain at risk of infection and their safety needs further study.

### 6.3 Fecal microbiota transplantation

Faecal microbiota transplantation (FMT) is a treatment that introduces flora or metabolites in donor faeces into diseased receptors to correct intestinal microecological imbalances and rebuild normal intestinal function. A meta‐analysis speculates that faecal transplantation may be more effective than probiotics in restoring intestinal flora, because faecal flora infusion can overcome the short‐term efficacy of probiotics and make a permanent change in flora.^105^ At present, FMT has achieved ideal efficacy in the treatment of recurrent or refractory Clostridium difficile infection. Recently, FMT has also been tested as an emerging therapy to manage cardiometabolic disorders. Vrieze et al^106^ performed FMT treatment in patients with metabolic syndrome and the results showed that insulin sensitivity and intestinal acid production in the faecal donor group were significantly elevated suggesting that FMT could be a potential therapeutic strategy for metabolic‐related diseases. In addition, in a randomized controlled trial, researchers transplanted the faeces of thinner people into fatter ones and 55% of the participants showed improved insulin resistance after 6 weeks.^107^ However, the use of FMT is currently limited because of its associated risks including the possible transfer of endotoxin or dangerous drugs that may cause new gastrointestinal complications. Further studies are needed to test whether FMT can extend to other aspects of cardiac metabolic disorders.^1^

### 6.4 Antibiotics

Antibiotic therapy has greatly disrupted the short‐term and long‐term microbial balance including reducing the richness and diversity of bacterial flora. Murphy et al^108^ fed mice with vancomycin and the results showed that the number of Firmicutes and Bacteroides decreased significantly, whereas the number of Proteus increased significantly. Tiihonen et al^109^ reported that the use of non‐steroidal anti‐inflammatory drugs affects the composition of intestinal flora in elderly samples and the use of anti‐inflammatory drugs can reduce the content of isobutyric acid, isovaleric acid and L‐lactic acid. But there are also studies showing that Vancomycin can change the intestinal flora richness, reduce MI area and improve the recovery of mechanical function after ischemia/reperfusion injury in rats.^110^ Polymyxin B and tobramycin can reduce the endotoxin content of Gram‐negative bacteria in the intestine and faeces and the contents of IL‐1beta, IL‐6 and TNF‐alpha in vivo in patients with HF.^111^ However, polymyxin B has been clinically restricted because of its toxicity. This situation suggests that we should pay attention to the side effects of antibiotics as well as their clinical effects. The improper use of antibiotics can kill beneficial bacteria in vivo, make pathogenic bacteria resistant and bring various adverse reactions. At present, how to clarify the mechanism of adverse reactions of antibiotics and maximize its clinical curative therapeutic effect is still an problem to be solved.

### 6.5 TMA inhibitors and other intervention methods

Research shows that choline structural analogues (3,3‐dimethyl‐1‐butanol) can block the choline metabolic pathway, reduce TMA production and play a role in the prevention and treatment of CVD.^112^ However, studies have shown that drugs that inhibit production of TMA‐FMOs can lead to acute hepatitis and fish odour syndrome.^68^ Another recent study shows that resveratrol can stimulate the growth of beneficial bacteria in the intestinal tract through the reconstitution of intestinal microflora, thus decreasing the production of TMAO.^113^ In addition, for colectomy, a study showed that patients with an average age of 45 years of age survived 1000 days after colectomy and compared with other five groups of non‐gastrointestinal surgery patients, colectomy did not reduce the risk of CVD and only reduced the risk of hypertension.^114^

## 7 CONCLUSION

Intestinal microecology is the largest microorganism system represented by intestinal microflora and the intestinal microflora together with the host maintains its microecological balance. Once the intestinal microflora is out of balance, it can quickly lead to a series of pathological and physiological changes including CVD. We have a new understanding of the pathogenesis of CVD and the prevention and treatment of CVD, especially for the development of new microecological agents in the clinical trial stage. At present, the research on intestinal microecology and CVD is mostly based on the basis and the internal mechanism is not fully understood. Therefore, the primary and clinical study of intestinal microecology and CVD needs to be further carried out, especially for guiding treatment of coronary heart disease and HF.

## CONFLICTS OF INTEREST

The authors confirm that there are no conflicts of interest.
